# Selecting and Characterizing Tyrosinase Inhibitors from *Atractylodis macrocephalae* Rhizoma Based on Spectrum-Activity Relationship and Molecular Docking

**DOI:** 10.1155/2021/5596463

**Published:** 2021-04-14

**Authors:** Yong-Qin Liu, Chang-Yan Xu, Fang-Yu Liang, Pei-Chun Jin, Zhi-Yao Qian, Zhong-Sheng Luo, Rong-Gui Qin

**Affiliations:** ^1^Pharmacy School, Guizhou Medical University, Guiyang, Guizhou, China; ^2^School of Public Health, Guizhou Medical University, Guiyang, Guizhou, China; ^3^The Key Laboratory of Chemistry for Natural Products of Guizhou Province and Chinese Academy of Sciences, Guiyang, Guizhou, China; ^4^State Key Laboratory of Functions and Applications of Medicinal Plants, Guizhou Medical University, Guiyang, Guizhou, China

## Abstract

*Atractylodis macrocephalae* Rhizoma (AMR) is a famous classical Chinese traditional medicine (CTM), which has been used as a tonic for many diseases for thousands of years. In ancient China, it was used as a supplementary food for beauty in the palace. In preliminary studies, the function of whitening skin and the significant inhibiting effect on tyrosinase (TYR) which is the reactive enzyme in the composition of melanin of AMR were discovered, and the relevant research was rarely reported. In this study, high-performance liquid chromatography (HPLC) along with partial least squares regression analysis (PLS) was applied to survey the coherence between the chemical constituents and the inhibiting activity of 11 batches of AMR on TYR activity. The results of PLS showed that the chromatographic peaks 11 (atractylenolide III) and 15 could be important effective ingredients of the inhibition TYR activity as ascertained by spectrum-activity relationships. Furthermore, TYR inhibitory activity of atractylenolide III was validated by in vitro test by *β*-arbutin served as a positive control drug. The results of the in vitro test and the molecular docking showed that atractylenolide III has high TYR inhibitory activity and could link to the residues in TYR catalytic pocket. Therefore, bioassay, molecular docking, and spectrum-activity relationships are appropriate for linking the quality of samples with pharmaceutical-related active ingredients. And our studying would lay a theoretical foundation for applying the water extracts of AMR in whitening cosmetics.

## 1. Introduction

Tyrosinase (TYR), also nominated as polyphenol oxidase, is a polyfunctional glycosylated copper-containing enzyme which exists in the organism of animal, plant, and microorganism widely [1–3]. In the presence of molecular oxygen, tyrosine is firstly catalyzed by TYR to oxidize to dopa, and then further oxidized to dopaquinone, which isomerized to form dopa pigment. Dopa pigment finally formed melanin with the participation of CO_2_ and TRP-2, which cause various skin diseases such as hyperpigmentation, melasma, freckles, and age spots [4, 5]. TYR plays a vital role because it is the critical enzyme and restriction enzyme in the course of melanin composition [6–8]. Pigment spots and melanoma markedly increased by increasing TYR activity and quantity [9]. Nowadays, TYR inhibitors have received broad attention due to their latent use as hypopigmented agents [10].

*Atractylodis macrocephalae* rhizoma (AMR), the dry rhizoma of *Atractylodes macrocephala* Koidz., is one of the Chinese herbal medicines compiled in Chinese Pharmacopoeia [11–15]. In ancient China, AMR was optioned to form the famous classic formula for whitening designated “Seven-White Ointment” and used as supplementary food for beauty in the palace [16]. It was reported that AMR mainly contained sesquiterpenoids and triterpenoid (including atractylenolideI, atractylenolide II, and atractylenolide III), polyacetylenes, coumarins and phenylpropanoids, flavonoids and flavonoid glycosides, polysaccharides, steroids, benzoquinones, and other constituents [17, 18]. However, its effects of whitening skin and active ingredients were rarely reported. The study of the spectrum-activity relationship was applied to explore their relevances.

The research of the spectrum-activity relationship can not only circumvent the shortcoming of segregation between chemical ingredients and pharmacodynamics but also sufficiently associate fingerprint with pharmacodynamics by the mathematical model [19, 20]. The spectrum-activity relationship explores the relevance between the fingerprint and pharmacodynamics to offer a credible method for clarifying the material base of Chinese herbal medicine [21]. The fingerprints were established by HPLC, UPLC, GC, GC-MS, and LC-MS usually [19, 22–24]. “Pharmacodynamics” data is acquired by the biomodels usually [25]. The methods of data processing, mainly include principal component analysis (PCA), partial least squares (PLS) regression analysis, orthogonal partial least squares-discriminant analysis (OPLS-DA), canonical correlation analysis (CCA), and grey relational analysis (GRA) usually [26–29].

To clarify the components of the AMR that contribute to the inhibition activity of TYR [30, 31], the fingerprints of 11 batches of AMR were established by HPLC; pharmacodynamic of TYR inhibition activity in vitro was evaluated by the biochemical enzymatic method. The effective compounds were selected by spectrum-activity relationship model which was established by correlated fingerprint peaks with pharmacodynamic data. Furthermore, active substances would be validated by in vitro test and molecular docking experiments. This study could lay a theoretical basis for applying AMR as the treatment medicine for pigmented skin diseases and developing it as whitening cosmetics supplementary.

## 2. Materials and Methods

### 2.1. Chemicals and Materials

Methanol (≥99.5%) was bought from Sinopharm Chemical Reagent Co., Ltd. (Shanghai, China). Phosphoric acid (≥99%) was purchased from Chengdu Jinshan Chemical Reagent Co., Ltd. (Chengdu, China). Acetonitrile was bought from Tiandi Reagent Co., Ltd. (USA). Atractylodes III (≥98%), phosphate buffer, tyrosinase, and L-tyrosine were bought from Beijing Soleibao Technology Co., Ltd. (Beijing, China). *β*-Arbutin (≥99.7%) was purchased from the China Academy of food and drug identification. Tyrosinase and L-tyrosine were dissolved in phosphate buffer (pH 6.8) before use. Atractylodes III and *β*-arbutin were dissolved in 50% methanol (v/v). The pure water was bought from Hangzhou Wahaha Baili Food Co., Ltd., (Hangzhou, China). Comix C18 column (250 × 4.6 mm, 5 *μ*m) was bought from Guangzhou Philomon Scientific Instrument Co., Ltd. (Guangzhou, China).

### 2.2. Plant Materials

The 11 batches of AMR medicinal samples were gathered and are shown in Table 1. The above samples were authenticated by Professor Ronggui Qin (School of Pharmacy, Guizhou Medical University).

### 2.3. Extraction

Herbal pieces of each sample (about 10 g) were weighed precisely and then placed in a round bottom flask, adding water and refluxing to extract. Products were dried under reduced pressure to obtain the water extract of AMR. Then, the water extracts of AMR (about 30.00 mg) were weighed precisely and dissolved with 50% methanol (v/v).

### 2.4. HPLC Analysis

Comix C18 reversed-phase chromatography column (250 × 4.6 mm, 5 *μ*m). The mobile phase 0.1% phosphoric was a mixture of acid water (*A*)-acetonitrile (*B*); the elution system is designed and is listed in Table 2. The flow rate was fixed at 0.6 mL/min. The column temperature was 30°C. The UA wavelength was 210 nm. The injection volume was 30 *μ*L.

The detection method was verified by precision test, repeatability test, and stability test.

### 2.5. Chemometric Analysis

In this study, the common peaks information of fingerprints of 11 batches of AMR were imported into software SIMCA14.1 for HCA, PCA, and OPLS-DA.

### 2.6. TYR Inhibition Test In Vitro

The extracts of AMR were dissolved with phosphate (pH 6.8) buffer, stored at 4°C, and then used for the assay of the enzyme.

In this study, L-tyrosine was used as a substrate to determine the inhibition of TYR activity. The reaction solutions were prepared according to Table 3, and the inhibition rate of AMR on TYR activity was determined. Firstly, the reaction solutions were put in a 37°C water bath for 10 min. Secondly, the TYR solution was added to T2 and T4 reaction solutions immediately. Thirdly, the reaction solutions reacted at 37°C water bath constant temperature for 10 min after mixed fully. Finally, the reaction solutions of the absorbance values were measured at 475 nm immediately. The inhibition rates (%) were obtained from the equation: the inhibition rate (%) = [(*A*_T2_ − *A*_T1_) − (*A*_T4_ − *A*_T3_)]/(*A*_T2_ − *A*_T1_) × 100%.

### 2.7. Spectrum-Activity Relationship Analysis

The software “Chinese traditional medicine chromatographic fingerprint similarity evaluation system 2012 A Edition” was used to adjust the retention times of each peak, and the peak area (PA) was processed by equalization. Then, the quantitative data were obtained. The PLS regression equation was set up with the software SIMCA14.1; the peak area was taken as the independent variable (*X*), and TYR inhibition rate was set as the dependent variable (*Y*).

### 2.8. Inhibitory Effect of Atractylodes III on TYR and Molecular Docking

To testify the inhibitory effect of Atractylenolide III on TYR, in vitro enzymatic activity tests were conducted by *β*-arbutin serving as a positive control drug.

The AutoDock4.2 program was applied in docking simulations. The crystal structure of *Agaricus bisporus* (PDB ID: 2Y9X) was taken as the 3D structure of TYR [32]. We performed simulations of the docking of TYR to Atractylodes III. With the purpose of docking with AutoDock Vina, the grid size was designed to (*x*, *y*, *z*) = (16, 12, 14) and the grid center was designed to (*x*, *y*, *z*) = (−10.348, −28.279, −45.925). In each simulation procedure, progress with default parameters operates from AutoGrid and AutoDock. Lamarckian genetic algorithm (LGA) was adopted to ascertain the most appropriate ligand binding orientations.

## 3. Results and Discussion

### 3.1. Methodology Validation

Precision, repeatability, and stability were validated for the analytical method. In precision testing, precision of relative retention times (RRTs) and relative peak areas (RPAs) did not exceed 0.02% and 4% in RSD, respectively; the similarity was 1. Repeatability of RRTs and RPAs was less than 0.11% and 4% in RSD, and the similarity was greater than or equal to 0.998. The stability was estimated by testing one sample solution preserved at lab environmental temperature after 0, 6, 8, 12, and 18 h. The RSDs of RRTs and RPAs of the common peaks were less than 0.1% and 4%, respectively; the similarity was more than 0.999. These results suggested that the AMR experimental system for fingerprint analysis is steady and dependable.

### 3.2. HPLC Fingerprint Establishment and Similarity Analysis

**Figure 1:**
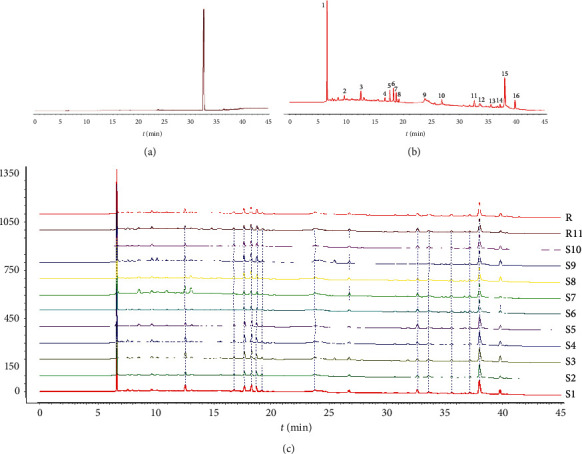
HPLC fingerprints of 11 batches of AMR. (a) Atractylodes III reference substance. (b) Shared mode. (c) Testing sample.

The fingerprint of Atractylodes III reference substances is exhibited in Figure 1(a). The AMR sample showed favorable segregation among its peaks (Figure 1(b)). Under the perfect conditions, the HPLC fingerprints of the 11 different batches of AMR samples were generated (Figure 1(c)), and the similarities were calculated. The results of similarities are shown in Table 4, which showed that all of the similarity values of the 11 samples were greater than 0.8, indicating that all samples were similar in the kinds of chemical compositions. Sixteen common peaks were observed by comparing their retention time of the UV spectrum. Peak 11 was identified as Atractylodes III by reference substances. The RSDs of PAs of 16 common peaks were between 1.18% and 58.68% (Table 5). These results indicated that the contents of the chemical substances in AMR from different production areas were obviously different.

### 3.3. Chemometric Analysis

#### 3.3.1. HCA

**Figure 2:**
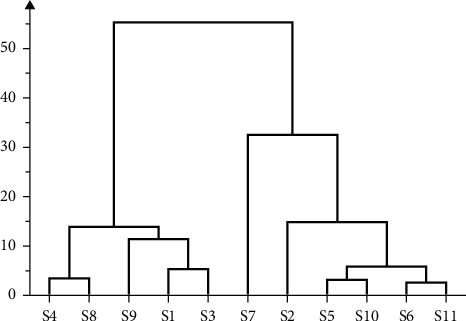
Cluster analysis of 11 batches of AMR.

HCA was applied to identify AMR from different production areas based on different clusters and the similarity of fingerprints. The common peak area in 11 batches of AMR was used as an indicator, and the cluster analysis was performed by software SIMCA14.1. The results are shown in Figure 2. The samples of 11 batches of AMR were divided into 3 categories when the class distances ranged between 20 and 30, of which S4, S8, S9, S1, and S3 were grouped into category I and S7 was grouped into category II, while S2, S5, S10, S6, and S11 were grouped into category III.

#### 3.3.2. PCA

**Figure 3:**
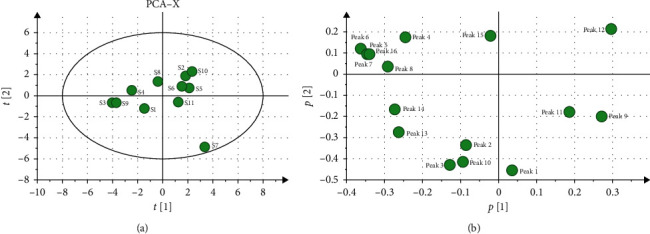
PCA analysis of 11 batches of AMR. (a) PCA scatter plot of 11 batches of AMR. (b) PCA load diagram of 11 batches of AMR.

In this study, the PCA of 11 batches of AMR was calculated; in 16 principal components, the cumulative contribution of the variance of the first five principal components was 91.3%. The score matrix of the first two components would be used for analysis as the cumulative variance exceeded 65.9%. Therefore, in the absence of some information, construct a two-dimensional plane of the principal component that the abscissa was a principal component and the ordinate was another principal component. Then, the 11 samples were projected onto the 2D plane so that their natural gathering was observed (Figure 3(a)). It was founded that S4, S8, S9, S1, and S3 had obvious classifications, S7 could be divided into one type, and S2, S5, S10, S6, and S11 could be divided into another type. The result of PCA was consistent with the HCA result.

Each dot in the load diagram represented a chromatographic peak, which represents the contribution of each chromatographic peak to the comprehensive effect of the principal components. The weight value of the variables can reflect the correlation between the chemical composition and the sample to the greatest extent. The farther away from the origin of the load diagram, the greater the variable weight. The result is shown in Figure 3(b), which suggested that the chromatographic peaks 9, 11 (atractylenolide III), and 12 had a greater impact on the first principal component. However, the chromatographic peaks 4, 5, 6, 15, and 16 had a greater impact on the second principal component. It showed that the above chromatographic peaks might have a greater impact on the classification.

#### 3.3.3. OPLS-DA

**Figure 4:**
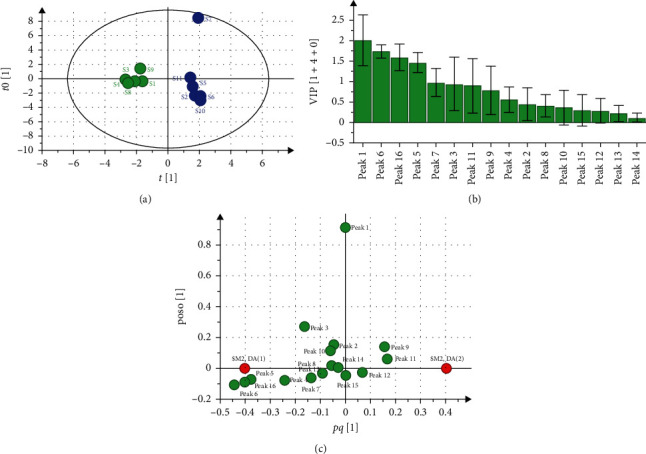
OPLS-DA analysis of 11 batches of AMR. (a) OPLS-DA scatter plot. (b) VIP values. (c) Load scatter plot.

The 11 samples were divided into two groups that S1, S3, S4, S8, and S9 were the first group and S2, S5, S6, S7, S10, and S11 were the second group. The common peak information of all samples was imported into SIMCA14.1 software for OPLS-DA. *R*^2^*X* and *R*^2^*Y* characterize the explanatory rate of the model to *x* and *y* matrices, individually, and *Q*^2^ characterizes the predictive ability of the model. The results showed that the values of *R*^2^*X*, *R*^2^*Y*, and *Q*^2^ were 0.939, 0.992, and 0.583, respectively, all of which were greater than 0.5, indicating that the model could distinguish the two groups of samples and had a better fitting and predictive ability in the data processing. It could be used to distinguish AMR from different production areas (Figure 4(a)).

The profile of variables important for the projection (VIP) in the OPLS-DA model can reflect the contribution of each chromatographic peak to the sample. The VIP values of the 16 chromatographic peaks reflected the influence power on every AMR sample. Based on the selection principle that the VIP value was more than 1.0 and the error bar was less than the origin (Figure 4(b)), the 4 important chromatographic peaks were selected out, which were arranged in order: peak 1 > peak 6 > peak 16 > peak 5.

The analysis results of the load diagram are shown in Figure 4(c), which showed that the positions of the above 4 important chromatographic peaks were far away from the origin. It was suggested that the 4 chromatographic peaks impacted the classification of AMR significantly, and these components represented by 4 chromatographic peaks might be the main marker components of each sample.

### 3.4. Assessment of Inhibitory Activity of AMR on TYR In Vitro

The inhibitory effects of AMR from different producing areas on TYR activity were determined by the biochemical enzyme method. The effects of samples on TYR activity are listed in Table 6. Each sample with 10 mg/mL concentration could inhibit TYR activity and pharmacological activity was remarkably different, among which the inhibition rate of S5 was the highest, whereas S6 was the lowest.

### 3.5. Spectrum-Effect Relationships Analysis

**Figure 5:**
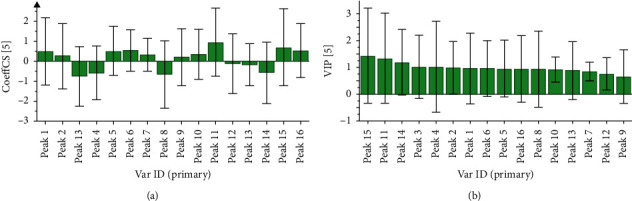
Correlation analysis between common peak area of the AMR and its inhibition rate of tyrosinase activity. (a) Normalized regression coefficient diagram. (b) VIP values.

In this article, PLS was applied to analyze the spectrum-activity relationship. The partial regression coefficient and VIP value are shown in Figures 5(a) and 5(b), which showed that chromatographic peaks 1, 2, 5, 6, 7, 9, 10, 11, 15, and 16 were correlated positively with the inhibition of TYR activity, but the chromatographic peaks 3, 4, 8, 12, 13, and 14 were negatively correlated with them. Among the chromatographic peaks positively correlated with the inhibition of TYR activity, the VIP values of chromatographic peaks 11 (Atractylodes III) and 15 were greater than 1, which indicated that these two components influenced the inhibition of TYR activity significantly.

### 3.6. Inhibitory Effect of Atractylodes III on TYR and Molecular Docking

The inhibitory effect of Atractylodes III on TYR activity was determined by biochemical enzyme method in vitro with *β*-arbutin as a positive control. The results showed that the inhibition rates of Atractylodes III and *β*-arbutin on TYR activity were 63.68 ± 2.36% and 94.85 ± 0.35%, respectively, when the samples' concentration was 1 mg/mL.

**Figure 6:**
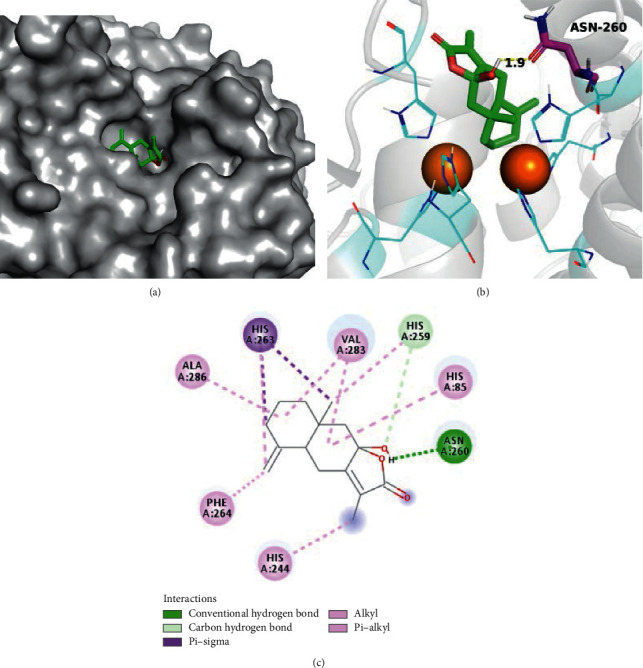
(a) Docking model of atractylenolide III in the mushroom tyrosinase ligand binding pocket. (b) 3D ligand-receptor interactions of atractylenolide III with the mushroom tyrosinase. (c) 2D ligand-receptor interactions of atractylenolide III with the mushroom tyrosinase.

Molecular docking was used to study the binding mechanism of Atractylodes III interacting with TYR (Figures 6(a)–6(c)). The binding site of mushroom tyrosinase is composed of a narrow, shallow cavity with two copper ions (Cu^2+^) each stabilised by three histidine residues (e.g., His 61) at the enzyme's active center. In this study, Atractylodes III is a small molecule compound located in the active center of the enzyme and interacts with the surrounding residues (His 244, Phe 264, Ala 286, His 263, Val 283, His259, His 85, Asn 260). Remarkably, Atractylodes III formed a stable hydrogen bond to Asn 260 with a bond length of 1.9 Å.

## 4. Conclusions

In this study, a systematic method was established connecting the HPLC fingerprints with chemometric analysis to distinguish AMR samples from different origins, and the whitening effect of AMR was proved by the biochemical enzyme method. Spectrum-effect relationships indicated that the chromatographic peaks 1, 2, 5, 6, 9, 10, 11, 15, and 16 were positively correlated with AMR inhibition effect on TYR activity, among which peaks 11 (atractylenolide III) and 15 might be important active constituents. Meanwhile, atractylenolide III has high TYR inhibitory activity in vitro test, and the result of molecular docking showed that its mechanism might be related to the binding of amino acid residues in TYR catalytic capsule. Therefore, these results proved that bioassay, molecular docking, and spectrum-activity relationships are appropriate for linking the quality of samples with pharmaceutical-related active ingredients.

## Figures and Tables

**Table 1:** Information about the collected AMR.

Number	Origin	Batch number
S1	Hubei	20190101
S2	Hunan	180601
S3	Sichuan	19050101
S4	Zhejiang	20190401
S5	Hebei	1908007
S6	Zhejiang	181201
S7	Hunan	1903105
S8	Zhejiang	18110102
S9	Sichuan	181104
S10	Zhejiang	19062502
S11	Hubei	1904005

**Table 2:** The time program of gradient elution.

Time (min)	*A* (%)	*B* (%)
0–12	90–70	10–30
12–17	70	30
17–20	70–42	30–58
20–23	42–40	58–60
23–27	40–30	60–70
27–32	30–15	70–85
32–35	15–5	85–95
35–45	5	95

**Table 3:** Composition of reaction liquid (company: *μ*L).

Sample	*T*_1_	*T*_2_	*T*_3_	*T*_4_
0.02% L-tyrosine	100	100	100	100
PBS	300	200	200	100
Sample solution	0	0	100	100
Tyrosinase solution	0	100	0	100

**Table 4:** 11 batches 0f AMR sample similarity evaluation.

Number	S1	S2	S3	S4	S5	S6	S7	S8	S9	S10	S11	Contrast fingerprint
S1	1.000	0.974	0.982	0.988	0.981	0.983	0.796	0.975	0.932	0.969	0.969	0.982
S2	0.974	1.000	0.949	0.958	0.971	0.988	0.755	0.962	0.895	0.990	0.954	0.964
S3	0.982	0.949	1.000	0.987	0.972	0.952	0.831	0.986	0.968	0.936	0.964	0.985
S4	0.988	0.958	0.987	1.000	0.975	0.970	0.825	0.986	0.956	0.947	0.973	0.987
S5	0.981	0.971	0.972	0.975	1.000	0.978	0.841	0.985	0.942	0.959	0.984	0.989
S6	0.983	0.988	0.952	0.970	0.978	1.000	0.762	0.961	0.904	0.984	0.964	0.969
S7	0.796	0.755	0.831	0.825	0.841	0.762	1.000	0.858	0.916	0.681	0.896	0.882
S8	0.975	0.962	0.986	0.986	0.985	0.961	0.858	1.000	0.966	0.942	0.980	0.993
S9	0.932	0.895	0.968	0.956	0.942	0.904	0.916	0.966	1.000	0.857	0.963	0.974
S10	0.969	0.990	0.936	0.947	0.959	0.984	0.681	0.942	0.857	1.000	0.924	0.940
S11	0.969	0.954	0.964	0.973	0.984	0.964	0.896	0.980	0.963	0.924	1.000	0.993
Contrast fingerprint	0.982	0.964	0.985	0.987	0.989	0.969	0.882	0.993	0.974	0.940	0.993	1.000

**Table 5:** 11 batches of AMR samples common peak information.

Peak number	*t*_R_ (min)	S1	S2	S3	S4	S5	S6	S7	S8	S9	S10	S11	RRT RSD (%)	RPA RSD (%)
Peak 1	6.620	10.565	8.111	13.276	12.917	11.528	8.416	36.269	13.539	20.265	5.847	14.694	0.25	58.68
Peak 2	9.643	1.246	1.175	1.728	1.979	1.571	1.129	2.306	1.437	2.245	1.437	1.952	0.04	25.26
Peak 3	12.542	4.416	2.262	4.605	3.358	2.031	1.528	5.389	1.923	3.348	1.988	2.345	0.04	42.95
Peak 4	16.750	1.447	1.112	1.624	1.857	1.316	1.234	0.977	1.510	1.223	1.406	1.255	0.06	18.08
Peak 5	17.630	3.942	2.800	5.235	5.444	3.393	2.923	2.270	4.777	4.383	3.175	3.145	0.05	28.01
Peak 6	18.249	5.042	3.519	6.590	6.371	4.059	3.437	2.119	5.797	6.246	3.850	3.757	0.06	31.89
Peak 7	18.758	3.640	3.204	3.948	3.649	2.466	2.641	1.970	3.227	4.269	2.854	3.487	0.06	21.25
Peak 8	19.246	1.205	1.233	1.291	1.258	0.805	1.070	0.783	0.894	1.579	0.960	1.225	0.07	21.57
Peak 9	23.801	2.960	3.207	1.778	3.012	2.642	2.408	4.352	2.941	1.702	2.942	3.171	0.06	25.59
Peak 10	26.749	2.223	1.626	2.239	1.528	1.557	1.658	2.268	1.625	1.733	1.350	1.787	0.03	17.82
Peak 11	32.644	3.569	1.465	1.976	2.390	4.204	2.947	3.392	1.855	1.167	2.287	3.747	0.02	37.91
Peak 12	33.610	2.371	2.462	2.311	2.367	2.449	2.373	2.383	2.371	2.254	2.480	2.357	0.02	2.76
Peak 13	35.588	1.379	1.262	1.352	1.280	1.234	1.278	1.300	1.278	1.333	1.219	1.277	0.02	3.74
Peak 14	37.167	1.165	1.144	1.164	1.175	1.151	1.154	1.151	1.140	1.163	1.130	1.139	0.02	1.18
Peak 15	37.997	15.766	16.490	15.791	15.794	15.594	15.655	15.626	15.739	15.827	15.739	15.573	0.02	1.59
Peak 16	39.807	4.336	2.337	5.824	4.489	3.834	2.666	1.746	4.792	5.106	3.292	2.386	0.02	35.52

**Table 6:** Inhibition of the AMR from different producing areas on tyrosinase activity.

Number	Inhibition rate (%)
S1	20.17 ± 1.38
S2	41.36 ± 2.64
S3	30.47 ± 2.22
S4	25.31 ± 1.80
S5	79.23 ± 0.93
S6	17.39 ± 3.21
S7	49.21 ± 2.54
S8	74.35 ± 1.67
S9	33.51 ± 2.35
S10	21.15 ± 3.11
S11	64.76 ± 1.02

## Data Availability

The data used to support the fndings of this study are available from the corresponding author upon request.
